# Replacing meat and dairy with plant-based alternatives in the Netherlands: trade-offs in environmental impacts and critical nutrient intake

**DOI:** 10.1007/s00394-026-03908-w

**Published:** 2026-02-16

**Authors:** Yinjie Zhu, Afke C. L. Politiek, Emely de Vet, Marga C. Ocké

**Affiliations:** 1 Consumption and Healthy Lifestyles Chair Group, Wageningen University & Research, Hollandseweg 1, 6706 KN Wageningen, The Netherlands; 2 National Institute for Public Health and the Environment, Antonie Van Leeuwenhoeklaan 9, 3721 MA Bilthoven, The Netherlands; 3 Global Nutrition Chair Group, Wageningen University & Research, Stippeneng 4, 6708 WE Wageningen, The Netherlands

**Keywords:** Dietary transition, Protein transition, Sustainable diet, Environmental impact, Public health nutrition

## Abstract

**Background:**

Plant-based protein sources are promoted as more sustainable alternatives to animal-based protein sources. The Dutch policy aims for 50% of dietary protein to come from plants, yet comprehensive evidence on the environmental and nutritional impacts of this transition is limited. We examined these impacts in the Dutch diet.

**Methods:**

Dietary intake data from Dutch adults (18–65 years) in the 2019–2021 National Food Consumption Survey served as the reference diet, derived separately for men (n = 585) and women (n = 600). Four replacement scenarios—“no meat and dairy”, “no meat”, “half meat”, and “no red meat”—were modeled by partially or completely substituting meat and/or dairy with plant-based alternatives, using weight- and energy-based replacements. Environmental impacts (greenhouse gas emissions (GHG), land use, water footprint) and 13 macro- and micro-nutrients status were assessed.

**Results:**

Replacement scenarios reduced GHG (11.3–39.3%) and land use (7.6–17.9%) but increased water footprint (3.6–60.2%). The 'half meat' scenario met the Dutch 50% plant-based protein target for both sexes while largely preserving nutrient intakes and adequacy, with only a slight increase in vitamin B6 inadequacy in women. All other scenarios had a more negative nutritional impact; for example, the “no meat” and “no red meat” scenarios increased the risk of vitamin B12 and B6 inadequacy and reduced total protein and saturated fat intake. Weight- and energy-based replacements yielded similar results.

**Conclusions:**

Replacing animal-based protein sources with plant-based alternatives reduces environmental impact, except for the water footprint. A 50% meat substitution while maintaining dairy intake generally preserves population-level nutrient adequacy.

**Supplementary Information:**

The online version contains supplementary material available at 10.1007/s00394-026-03908-w.

## Introduction

Increasing attention has been given to the environmental impacts of diets in research, practice, and policy-making [1], while sustainable diets, as defined by the Food and Agriculture Organization (FAO), should also be healthy [2]. According to FAO and the World Health Organization (WHO), the nutrition and health dimension is at the core of sustainable diets, and the population’s demand for adequate nutrition and health could also impact the whole dietary transition [3]. Therefore, research related to the impact on nutritional quality and nutrients supply of dietary transition is of equal importance as the environmental impacts.

In the Netherlands, both the environmental impacts and nutrient supply have been addressed in a report published in 2023 by the Dutch Health Council (GR), where transitioning towards a diet with more plant-based protein is advised to achieve a healthy protein transition [4]. Currently, plant-based protein accounts for around 40% of the Dutch diet, and the aim of the Dutch government is to increase the proportion to 50% by 2030 [4]. It remains unclear how this transition would unfold at the population level, as well as how policymakers and intervention experts would communicate and implement it; furthermore, the impacts of such a dietary transition on public health nutrition are not evident [5]. With a priori scenarios of hypothetical diets, it can be investigated how we can reach the targeted plant-based protein consumption and evaluate the subsequent nutritional and environmental impacts [6, 7].

Despite the existence of other methodologies to study the sustainability of diets, i.e., design of more sustainable diets with constrained optimization [8], results from hypothetical diets are generally straightforward and easy to understand and communicate. We chose to replace animal-based food products with plant-based alternatives, with a focus on meat and dairy products, as they account for half of the daily protein intake in the Dutch population [9]. A handful of studies in Dutch contexts also applied similar hypothetical replacement scenarios for different populations (children, older adults, young adult females, and general adults) with a focus on various nutrient intakes and environmental impacts [6, 7, 9–12]. Generally, a full replacement of meat and dairy products yielded more environmental gains, together with certain nutrient inadequacies, especially for older adults and children, and partial replacement of meat and dairy resulted in less profound environmental gains but also less nutritional compromise [6, 7, 9–12]. Internationally similar results were also reported from various dietary scenario modelling [13, 14], which bear relevant implications to public health policy targeting sustainable dietary and nutrition transition.

Nevertheless, none of the studies investigated the effects of replacement on plant- and animal-based protein, other nutrients, and environmental impacts simultaneously. When simulating the replacement scenarios, previous literature has predominantly replaced the observed foods with an equal quantity of replacement foods, although replacement foods generally contain more plant-based components and therefore provide fewer calories per unit. It has been well-documented that energy compensation is present for diet-induced energy deficits at various magnitudes, which suggests an increased drive to eat to maintain total energy intake [15]. To our knowledge, only one study has compared the protein and amino acid intakes while applying equal energy supply replacements in those hypothetical diets [14], but it has not extended the analysis to other nutrients or environmental impact indicators.

In this study, we aimed to apply two replacement strategies, i.e., equal quantity and equal energy supply, to explore the extent to which the replacements of meat and dairy products in the current Dutch diets can reach the targeted 50% of plant-based protein consumption at the population level. More importantly, we also investigate the associated nutritional and environmental impacts of those hypothetical replacement scenarios.

## Methods

### Study population and observed reference consumption

The Dutch National Food Consumption Survey (DNFCS) is a national recurring survey that aims to gain insight into the dietary consumption of individuals living in the Netherlands. The recruited population represents the Dutch population as a whole, regardless of nationality, with the exception of pregnant and lactating women, people who were institutionalized, and those without adequate command of the Dutch language. The current study used the observed food consumption data from the latest DNFCS from 2019 to 2021 as the reference. Only the adult population aged between 18 and 65 was selected, as similar research has already been conducted on other age groups [6, 11], resulting in 585 men and 600 women. The consumption data of each participant were collected by means of a general questionnaire and through two non-consecutive 24-h dietary recalls, with an interval of about 4 weeks, using the computer- directed interview program GloboDiet by a trained interviewer [16]. Overall, all days of the week were equally represented in the recalls. The consumed amount of each food per participants were recorded in the dataset. The 24-h dietary recall (GloboDiet) used in DNFCS is an internationally standardized and validated methodology [17]. A detailed description of DNFCS 2019–2021 can be found elsewhere [18].

### Food composition data and replacement scenarios

After calculation of the consumed quantity of foods in DNFCS 2019–2021, the total energy intake, as well as the macro- and micro-nutrient intake, were calculated using the Dutch Food Composition Database (NEVO) version 2021/7.0 [19, 20]. The NEVO database contains data on the composition of foods that are frequently consumed by a large part of the Dutch population. The NEVO database is updated every few years to include new food products consumed in the Dutch population. In the latest version from 2023/8.0 [21, 22], 142 new foods were added because new foods were put on the market, consumption of the food was considerable, or new food composition data was available. For instance, a considerable number of new meat and dairy substitutes were added in the latest NEVO table. Thus, in our protein transition scenarios, replacement foods were chosen from the NEVO Table 2023. As mentioned in the introduction, we have chosen to replace meat and dairy products with their plant-based alternatives. The replacements were done in subgroups of meat and dairy products with plant-based alternatives that have similar functions in the diet. A brief overview of food groups to be replaced and replacement food groups can be found in Table 1. The number of replacement alternatives for each food group had an average of 12 (ranging from 1 to 31) items to ensure the randomness and representativeness of the dietary scenario.

**Table 1:** Overview of food groups with example foods as consumed in DNFCS 2019–2021 and corresponding plant-based replacement food groups

Consumed meat and dairy products in reference	Plant-based alternatives
*Dairy products*	
Milk drink, non-sweetened	Plant-based milk drink, non-sweetened
-e.g., whole milk, butter milk, skimmed milk	-e.g., soy milk, coconut milk, rice drink
Milk drink, sweetened	Plant-based milk drink, sweetened
-e.g., chocolate milk, yoghurt drink, fruit milk	-e.g., sweetened soy drink, chocolate soy drink
Coffee milk	Plant-based coffee creamer
Dairy dessert	Plant-based dessert
-e.g., vla, pudding, ice cream, mousse	-e.g., soy-based dessert, plant-based alternative to ice cream based on coconut
Fermented dairy products	Plant-based fermented protein products
-e.g., yoghurt, quark, kefir	-e.g., soy-based yoghurt
Cream products	Plant-based alternative products to cream
-e.g., Whipped cream, crème fraiche, cooking cream	-e.g., plant-based alternative to cream based on soy, oat
Cheese	Plant-based cheese
-e.g., brie, gouda, cheese 20 +	-e.g., plant-based alternative to gouda cheese based on coconut oil, vegetarian cheese
	Nuts -e.g., walnuts, cashew, nuts mix
Cheese spread	Plant-based savory spread
-e.g., cheese spread, dairy spread	-e.g., peanut butter, hummus, vegetarian pate
Cheese-based warm snacks	Plant-based savory warm snacks
-e.g., puff pastry with cheese	-e.g., vegetarian nuggets, vegetarian croquette
Meat products*	
Meat unprocessed	Plant-based meat substitute
-e.g., pork, chicken, beef, minced meat	-e.g., tofu, falafel, vegetarian minced meat
	Legumes
	-e.g., chickpeas, kidney beans, lentils
Meat processed	Plant-based processed meat substitutes
-e.g., sausages, salted beef, chicken schnitzel	-e.g., vegetarian schnitzel, vegetarian burgers, vegetarian sausages
Meat-based warm snacks	Plant-based warm snacks
-e.g., croquetten, bitterballen, frikandel	-e.g., vegetarian nuggets, vegetarian croquette
Cold meat spread	Plant-based savory spread
-e.g., pate, salad ham spread, salad chicken spread	-e.g., peanut butter, hummus, vegetarian pate
Cold meat cut	Plant-based cold meat cut alternatives
-e.g., salami, chicken filet cut, bacon	-e.g., vegetarian luncheon meat
	Nuts

*Red meat is a subgroup of the “meat products” category that includes meat products from cow, pig, horse, deer, sheep, and goat

The nutritional and environmental impacts of four replacement scenarios were chosen to be compared with those of the reference scenario, i.e., reference (current) consumption (Table 2). Comparisons between replacement scenarios were conducted by evaluating the relative changes in outcomes against the reference scenario; as this is a deterministic simulation model, no statistical tests were applied [7, 10–12]. Besides an extreme scenario “no meat and dairy” where both dairy and meat products were replaced with corresponding plant-based alternatives, the rest of the scenarios focused on the replacement of meat or red meat consumption only while remaining reference dairy consumption (“no meat”, “half meat”, and “no red meat”) because GR recommended no changes to existing consumption of dairy products due to its potential health benefits [23].

**Table 2:** Overview of the reference consumption and replacement scenarios

Scenarios	Description
Reference	The consumption observed from the DNFCS 2019–2021
No meat and dairy	All meat and dairy products consumed were replaced by plant-based alternatives
No meat	All meat products consumed were replaced by plant-based alternatives; while dairy products consumed stayed the same as the reference
Half meat	50% of the meat products consumed were replaced by plant-based alternatives; while dairy products consumed stayed the same as the reference
No red meat	All red meat products consumed were replaced by plant-based alternatives; while other meat and dairy products consumed stayed the same as the reference

*DNFCS: the Dutch national food consumption survey

Two types of replacement strategies were implemented, namely quantity replacement and energy replacement. In the quantity replacement strategy, for all foods, the amount in grams of the reference (current) consumption was replaced by exactly the same amount of foods from the corresponding replacement food groups. In the energy replacement strategy, for all foods, the amount of each replacement food was adjusted to provide the same number of calories in kcal as the reference foods. In both replacement scenarios, for each replacement of food, the probability of foods from the corresponding replacement food groups chosen as replacers was equal. The random allocation of the foods in replacement food groups was repeated ten times for all scenarios, and the outcomes of interest between these replicates were rather small (Coefficient of variation < 0.01). Thus, we have decided to choose a fixed seed for the allocation of every scenario [11].

### Habitual nutrient intakes and dietary reference intakes

For both reference and replacement scenarios, the nutrient intakes and environmental impact were calculated and compared. From the wide range of nutrients included in the NEVO database, total energy, total protein, plant-based protein, animal-based protein, total lipids, saturated fatty acids (SAFA), fiber, vitamin A (as retinol activity equivalents), B12, B6, and B2, calcium, and sodium. These nutrients were chosen because the GR advisory report “A Healthy Protein Transition” prioritized them as requiring public health attention and continued monitoring during the shift toward a more plant-based diet in the Netherlands. Other micronutrients such as iron, zinc, and vitamin D were not prioritized, as their relevance and monitoring needs in the Dutch population are already well-established in the existing literature [4]. The consumption data were collected by two non-consecutive 24 h recalls per person in the original DNFCS 2019–2021; statistical modelling that accounts for intra-individual variation was applied for estimating long-term habitual dietary intake.

The statistical program to assess dietary exposure (SPADE, version 4.1.44) was developed by the RIVM and implemented in R and is freely available as an R package called SPADE.RIVM [24]. The habitual intake distribution is modeled as a function of age for men and women separately, and this distribution can directly be compared with cutoff values to estimate the proportion above or below. Uncertainty in the habitual intake distribution and in the proportion below or above a cutoff value is quantified with a bootstrap and provides 95% CIs. The habitual intake distribution for all nutrients of interest in this study was estimated using the one-part model for components consumed on a daily basis by all subjects. A bootstrap of 200 was performed to account for uncertainties [25].

The adequacy of specific nutrients on a population level of reference and replacement scenarios was compared with the Estimated Average Requirement (EAR) [26, 27]. In practice, if more than 10% of a population has an intake below the EAR, this indicates a potential public health concern. The proportion of men and women with intakes below the EAR was calculated for micronutrients with available EAR, i.e., vitamin A, B12, B6, and B2, and calcium (Supplementary Table S1), which was set by the GR [28, 29].

### Environmental impacts

To calculate the environmental impact, the environmental-oriented life cycle (LCA) assessment dataset was used, where the life cycle of a food product is quantitatively analyzed and considered within the context of environmental impact. LCA was performed by RIVM using the latest updated life cycle inventory data from Mérieux NutriSciences | Blonk in 2023 (version 3.0) [30, 31]. The LCA data were developed for the purpose of monitoring the environmental impact of the Dutch food consumption [31]. In short, LCA is a method for assessing the environmental impacts associated with all stages of the life cycle of a product, from raw material extraction (cradle) to disposal (grave). The 2023 LCA data contains information for 411 foods covering 75% of the foods consumed in DNFCS 2019–2021, and extrapolations were made to all other foods existing in DNFCS 2019–2021 and NEVO 2023/8.0 by expert judgment based on similarities in types of food, origin, cultivation method, production systems, and ingredient composition [32, 33]. A detailed description of LCA used in this study can be found elsewhere [31, 34].

The life cycle assessment data were combined with the consumption data (in reference and replacement scenarios) to calculate environmental indicators. For this study, three indicators were selected for the evaluation of environmental impacts: greenhouse gas emission (GHG) emission or climate change (kg CO2-eq), land use (m^2^·year), and water footprint (liters). GHG emission indicates the global warming potential that covers CO_2_ emissions through the use of fossil fuels, as well as CH_4_ and N_2_O emissions expressed as kg CO2 equivalents (CO2-eq). Land use indicates the average use and transformation of land area for the production of food over a one-year time frame, expressed as square meters multiplied by year (m^2^·year). Water footprint is indicated as the volume of freshwater consumed in liters as a result of human activity, such as irrigation during crop cultivation, and is based on country-specific water requirement ratios to estimate actual irrigation water consumption [31].

### DISC-tool and statistical relevance

A new simulation model was developed by the RIVM that harmonized the DNFCS, NEVO, EAR of nutrients, LCA, and a framework to apply your scenario and replacement strategies as inputs of the model. This Dietary Impact Scenario (DISC) tool is implemented in R that harmonizes SPADE to estimate mean habitual intakes, with consumption data weighted by seasons and time of the week and LCA database including average values over all seasons (internal documentation, unpublished). A more detailed description of the DISC tool will be provided in a forthcoming publication. Currently, internally available at RIVM as an R Shiny web application or as the code, the DISC tool calculates the impact on nutrient intakes and environmental impact of three types of scenarios and combinations of those: adjusting the environmental impact of food products, adjusting the composition of food products, and replacing/reducing/increasing food product consumption. The current study chose to define various replacement scenarios. For each replacement scenario, the mean habitual energy and nutrient intakes with 95% CI are shown. The DNFCS weighting factor was included to account for deviances in sociodemographic characteristics, days of the week, and season of data collection, to make the results representative of the Dutch adult population. The 95% CI was computed using the bootstrap method with 200 iterations. Differences in habitual nutrient intakes and proportions below EAR among scenarios were evaluated by nonoverlapping 95% CI from the bootstrap, as indicated by previous literature [10, 11]. All analyses of habitual nutrient intakes were conducted separately for men and women, while the environmental impact analyses were done for the total population, as well as separately for men and women.

## Results

### Protein intake

The reference total protein intake was 71.0 g/day for women and 95.4 g/day for men (Supplementary Table S2). In the reference diet, total dairy intake was 388.1 g/day in women and 471.2 g/day in men, while total meat intake was 115.9 g/day and 176.2 g/day, respectively, of which red meat accounted for 91.8 g/day in women and 143.4 g/day in men (Supplementary Table S3). Total protein intake decreased in all replacement scenarios, except for "half meat" and "no red meat" under equal energy replacement (Figure S1). As expected, all scenarios reduced animal-based protein intake while increasing plant-based protein intake (Figure S2).

**Fig. 1:**
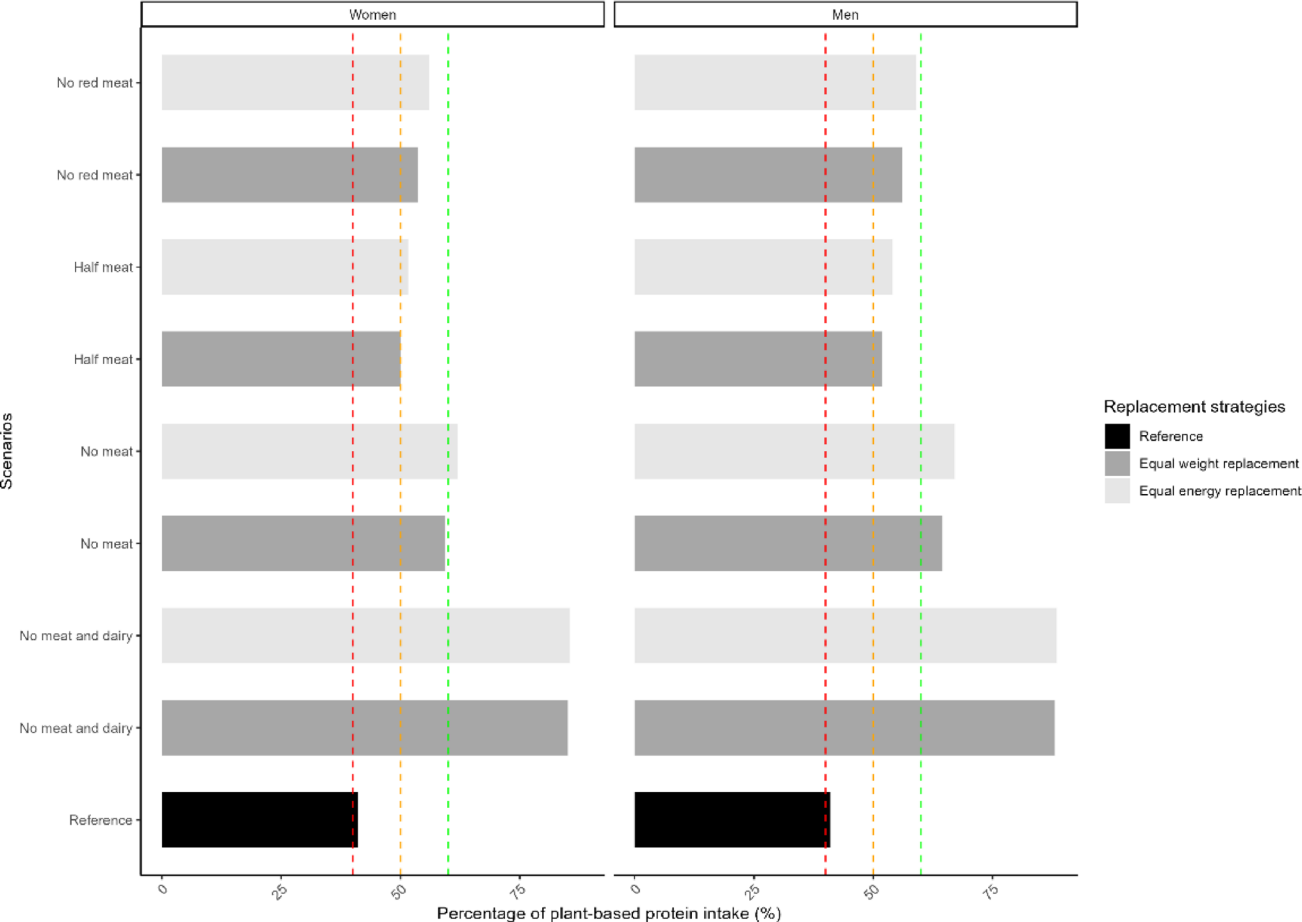
Percentage of plant-based protein intake (%) of all scenarios among women (left) and men (right). The red, orange, and green vertical dash lines represent the current (~ 40%), short-term target (50%), and long-term target (60%) consumption of percentage of plant-based protein intake (%) in the Dutch population

Currently, plant-based protein accounts for approximately 40% of total protein intake in both women (41.1%) and men (41.0%) (Fig. 1). Across all scenarios, the proportion of plant-based protein increased, reaching the highest levels (around 85% in women and 88% in men) with “no meat and dairy”. The “half meat” scenario achieved approximately 50% plant-based protein, aligning with national targets. In the “no meat” scenario, plant-based protein comprised ~ 60% of intake in women and over 60% in men, with minor variations between equal weight and equal energy replacement strategies. The “no red meat” scenario resulted in over 50% plant-based protein for both sexes, with the highest proportion (59.0%) in men under equal weight replacement (Fig. 1).

### Micronutrients and population reference intake

**Fig. 2:**
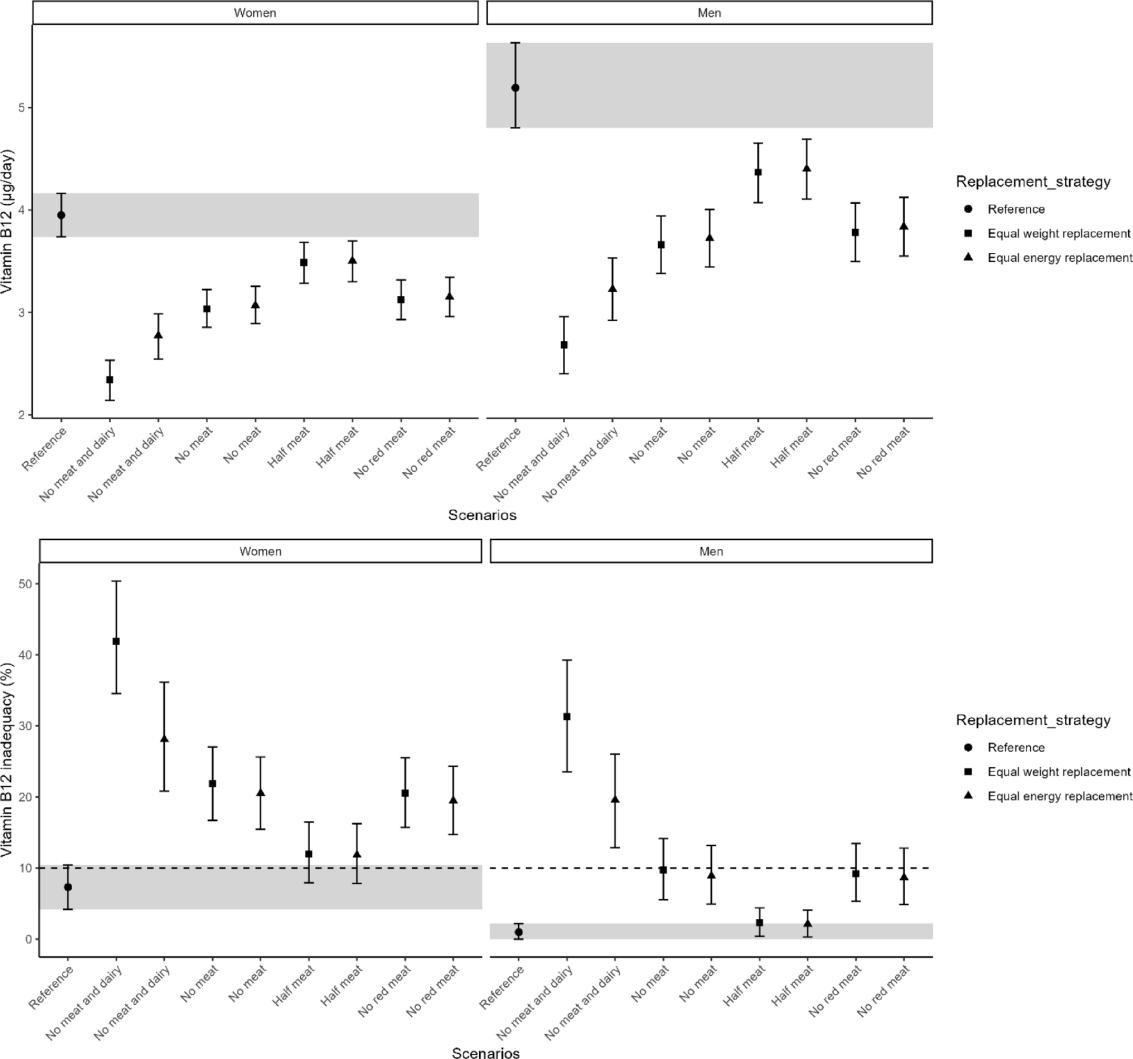
Habitual intake of vitamin B12 in μg/day (upper) and the percentage of the population at risk of vitamin B12 inadequacy (lower), indicated by the proportion below the estimated average requirement, are shown for the reference and replacement scenarios in women and men. The intervals lines represent 95% confidence interval derived from 200 bootstrap resamples for each scenarios. The horizontal dashed line indicates the cutoff for potential public health concern (10%), as applied by the Dutch National Institute for Public Health and the Environment (RIVM)

**Fig. 3:**
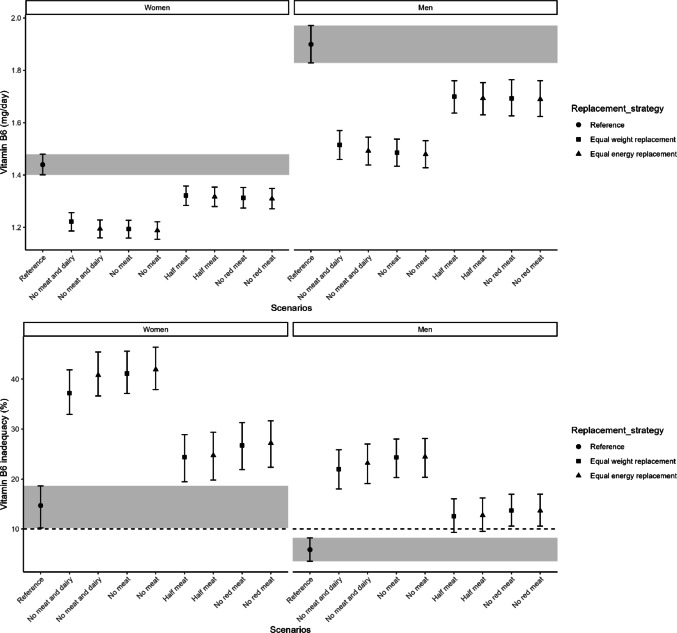
Habitual intake of vitamin B6 in mg/day (upper) and the percentage of the population at risk of vitamin B6 inadequacy (lower), indicated by the proportion below the estimated average requirement, are shown for the reference and replacement scenarios in women and men. The intervals lines represent 95% confidence interval derived from 200 bootstrap resamples for each scenarios. The horizontal dashed line indicates the cutoff for potential public health concern (10%), as applied by the Dutch National Institute for Public Health and the Environment (RIVM)

**Fig. 4:**
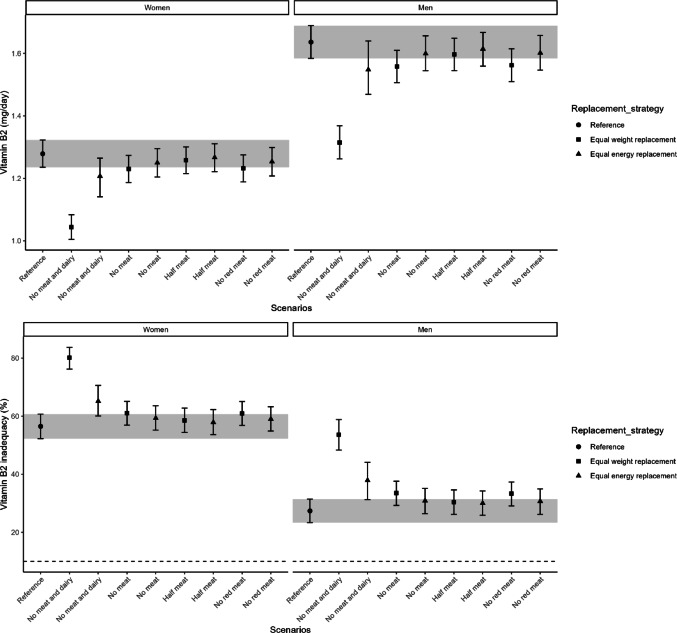
Habitual intake of vitamin B2 in mg/day (upper) and the percentage of the population at risk of vitamin B2 inadequacy (lower), indicated by the proportion below the estimated average requirement, are shown for the reference and replacement scenarios in women and men. The intervals lines represent 95% confidence interval derived from 200 bootstrap resamples for each scenarios. The horizontal dashed line indicates the cutoff for potential public health concern (10%), as applied by the Dutch National Institute for Public Health and the Environment (RIVM)

Habitual vitamin B12 intake decreased in all replacement scenarios compared to the reference intake of 3.9 [95% CI 3.7–4.2] µg/day in women and 5.2 [95% CI 4.8–5.6] µg/day in men (Supplementary Table S2). The largest reductions were observed in “no meat and dairy” scenarios, followed by “no meat” and “no red meat” (Fig. 2). Except for “half meat” scenarios, the risk of vitamin B12 inadequacy increased, exceeding 10% in women for most scenarios and in men only for “no meat and dairy” (Fig. 2 and Supplementary Table S4). Vitamin B6 intake (1.4 [95% CI 1.4–1.5] mg/day in women, 1.9 [95% CI 1.8–2.0] mg/day in men; Supplementary Table S2) declined across all scenarios, with the greatest reductions in “no meat and dairy” and “no meat” scenarios. This led to increased inadequacy risk, particularly in women, where over 20% of the population was at risk in all replacement scenarios (Fig. 3 and Supplementary Table S4). For vitamin B2 (1.3 [95% CI 1.2–1.3] mg/day in women, 1.6 [95% CI 1.6–1.7] mg/day in men; Supplementary Table S2), intake remained largely unchanged except for a decrease in the “no meat and dairy” equal weight scenario. The risk of inadequacy was comparable to the reference scenario, except for an increase in “no meat and dairy” scenarios for both sexes. (Fig. 4 and Supplementary Table S4).

**Fig. 5:**
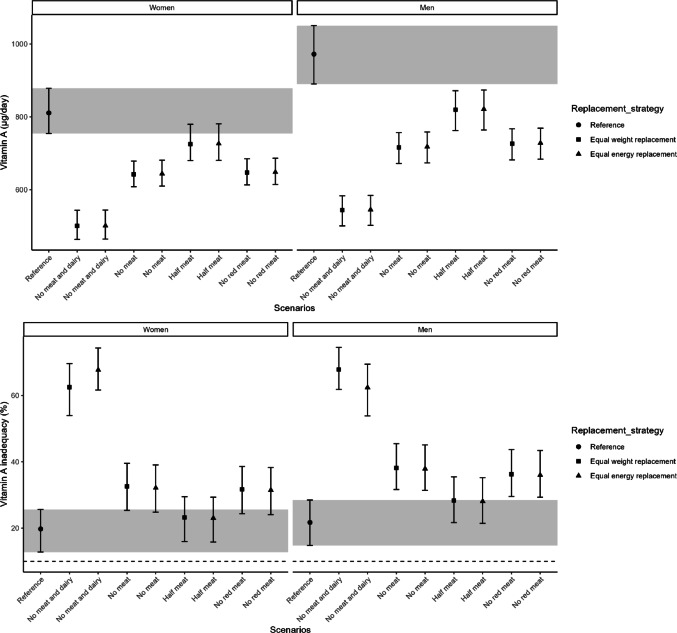
Habitual intake of vitamin A in μg/day (upper) and the percentage of the population at risk of vitamin A inadequacy (lower), indicated by the proportion below the estimated average requirement, are shown for the reference and replacement scenarios in women and men. The intervals lines represent 95% confidence interval derived from 200 bootstrap resamples for each scenarios. The horizontal dashed line indicates the cutoff for potential public health concern (10%), as applied by the Dutch National Institute for Public Health and the Environment (RIVM)

**Fig. 6:**
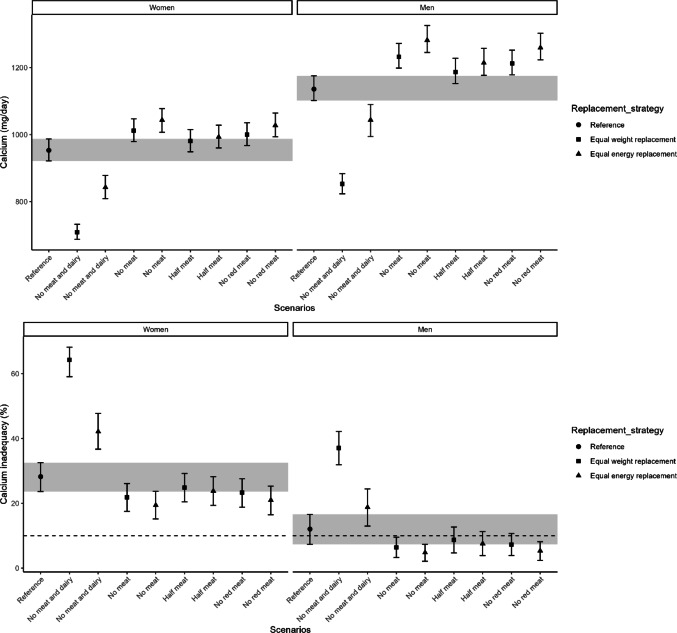
Habitual intake of Calcium in mg/day (upper) and the percentage of the population at risk of Calcium inadequacy (lower), indicated by the proportion below the estimated average requirement, are shown for the reference and replacement scenarios in women and men. The intervals lines represent 95% confidence interval derived from 200 bootstrap resamples for each scenarios. The horizontal dashed line indicates the cutoff for potential public health concern (10%), as applied by the Dutch National Institute for Public Health and the Environment (RIVM)

Vitamin A intake (811 [95% CI 754–878] µg/day in women, 972 [95% CI 890–1050] µg/day in men; Supplementary Table S2) decreased in all replacement scenarios except “half meat” in women. “No meat and dairy” had the largest decline, leading to increased inadequacy risk. In men, all scenarios except “half meat” resulted in a higher prevalence of inadequacy, while in women, only “no meat and dairy” increased risk (Fig. 5 and Supplementary Table S4). Calcium intake (953 [95% CI 921–987] mg/day in women, 1136 [95% CI 1102–1175] mg/day in men; Supplementary Table S2) decreased only in “no meat and dairy” scenarios, while other scenarios showed minimal change. The risk of calcium inadequacy increased in “no meat and dairy” scenarios for women and only in the “no meat and dairy” equal weight replacement for men, with no major differences elsewhere. Over 10% of women were already at risk in the reference scenario, while in men, only “no meat and dairy” scenarios exceeded this threshold (Fig. 6, Supplementary Table S4).

### Energy intake and other nutrients

Total habitual energy and lipid intake remained comparable across replacement scenarios, except for a higher intake of energy and lipid in the “no meat and dairy” equal weight replacement (Supplementary Figures S3 and S4). SAFA intake decreased in “no meat” and “no red meat” scenarios, as well as in the “no meat and dairy” equal energy replacement (Supplementary Figure S5). All replacement scenarios led to increased dietary fiber intake (Supplementary Figure S6). Sodium intake decreased in “no meat and dairy” for both replacement strategies and in “no meat” and “no red meat” under equal weight replacement (Supplementary Figure S7).

### Environmental indicators

**Fig. 7:**
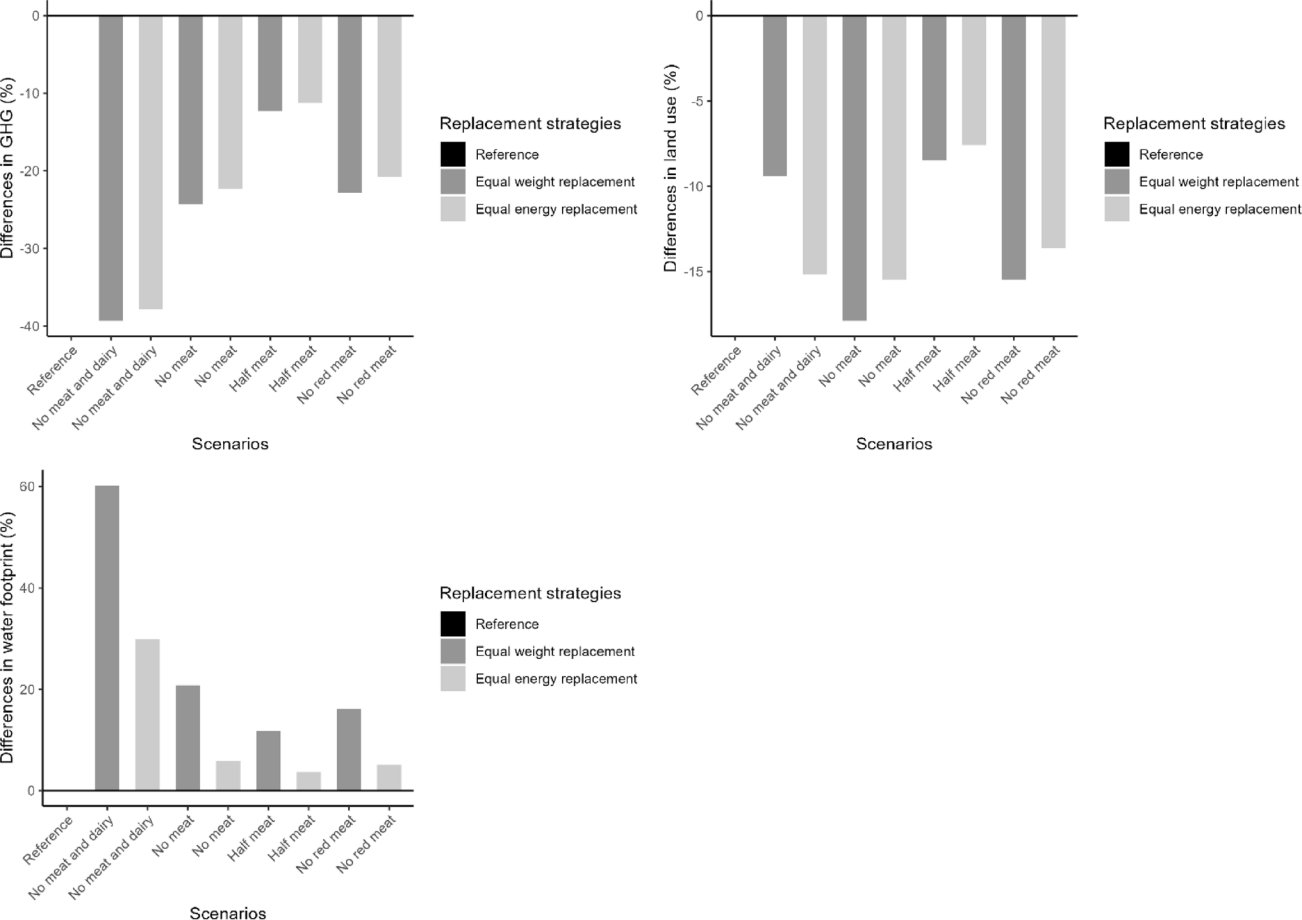
Relative differences of GHG (kg CO_2_-eq/day), land use (m^2^·year/day), and water footprint (liters/day) of replacement scenarios with reference scenario

In all replacement scenarios, GHG emissions and land use decreased, while water footprint increased (Fig. 7, Supplementary Figure S8, Supplementary Table S5). The reference diet had an average GHG footprint of 3.99 kg CO_2_-eq/day, land use of 3.30 m^2^·year /day, and water footprint of 95.82 L/day. Meat and dairy products contributed 47% and 28% of GHG emissions, 44% and 15% of land use, and 13% and 18% of water footprint, respectively (Supplementary Table S6). The greatest GHG reduction occurred in the “no meat and dairy” scenarios (2.42–2.48 eq_kg/day), followed by “no meat,” “no red meat,” and “half meat” scenarios, with the last showing the smallest reduction (3.50–3.54 eq_kg/day). Land use declined by 7.6% to 17.9%, with the largest decrease in the equal weight "no meat" scenario. In contrast, water footprint increased across all scenarios (3.6%–60.2%), with the highest rise in “no meat and dairy” (up to 153.55 L/day in equal weight replacement). Equal weight replacement led to a greater increase in water footprint than equal energy replacement for the same scenario.

## Discussion

We compared the environmental and nutritional impacts of four dietary replacement scenarios with the current Dutch diet as the reference scenario, where the meat and/or dairy products were partially or completely replaced with their plant-based alternatives through equal weight and equal energy replacement strategies. All replacement scenarios had reduced GHG emissions and land use, but increased water footprint, compared to the reference diet. Among the scenarios, the 'half meat' scenario represented the most moderate degree of replacement, with only half of the meat being replaced (dairy consumption remains unchanged), achieving the ‘protein transition’ policy goal of 50% plant-based protein while maintaining overall nutrient intakes. In this scenario, most nutrients remained adequate at the population level, with only slightly increased risk of inadequacy in vitamin B6 in women. Other scenarios with greater degrees of replacement achieved a higher proportion of plant-based protein intake but also led to a greater risk of micronutrient inadequacy and more substantial deviations in macronutrient intakes compared to the ‘half meat’ scenario.

Our results showed that for both sexes, the greater the degree of replacement, the higher the proportion of plant-based protein intake. The reference scenario showed that currently, the total protein intake for both men and women exceeded the recommended level of intake, i.e., approximately 50 to 60 g of protein per day for a Dutch adult with an average weight of 62–75 kg [35]; on the other hand, the current Dutch diet consists of approximately 40% plant-based protein. The national goal is to reach a 50/50 ratio of plant- and animal-based protein by 2030, adapted from the original target of 60/40 [4]. Notably, even the least extensive replacement scenario, where meat was partially substituted with plant-based alternatives while dairy intake remained constant (“half meat” scenario), already meets this 50/50 protein transition target. Furthermore, fully replacing meat (“no meat” scenario) shifts the diet toward the more ambitious 60% plant-based protein goal. These findings suggest that achieving the protein transition target at the population level in the Netherlands is feasible, particularly through moderate dietary changes via the “half meat” scenario. While this study quantified protein intake at the population level, it did not evaluate protein quality, such as the adequacy of indispensable amino acids, which merits further investigation in the context of a transition toward plant-based diets.

All replacement scenarios led to lower GHG and land use but increased water footprint. The reduced GHG and land use of diets with more plant-based alternatives have been reported in previous studies [7, 10, 13, 14, 36–38], although direct comparisons are challenging due to differences in choices of dietary scenarios, datasets, and population groups. Additionally, this tendency is observed not only in studies based on dietary scenarios but also in reports with real diets rich in plant-based products [39, 40]. However, the impact on water footprint when shifting towards more plant-based products has not been as extensively studied and remains more uncertain than for GHG emission and land use, partly because water footprint assessment methodologies vary in scope, system boundaries, and underlying data availability [41, 42]. For example, the water footprint in this study did not take into account the local capacity of reservoirs. These methodological inconsistencies contribute to the limited comparability and interpretation of water footprint results across dietary studies [42]. While we observed increased water footprint with the increase of the plant-based protein sources, studies also reported a reduction of (fresh) water use [14, 37]. For example, a modelling study conducted in a Swedish context found that diets rich in plant-based alternatives, including vegan, vegetarian, and flexitarian scenarios, moderately reduced freshwater use (14–27%) [14]. On the other hand, a global modelling analysis with country-level detail showed that the freshwater use increases of up to 16% in high-income countries by replacing animal-source food with plant-based ones [13]. The higher water demand from our scenarios is likely due to water-intensive plant-based protein sources, such as legumes and nuts. It has been reported that the water used to obtain calorie-equivalent amounts of nuts and legumes could be higher than that of several animal-based products [43]. Thus, while increasing the consumption of plant-based protein sources is often considered a key indicator of sustainable diets, it does not capture all environmental aspects. Similarly, a recent study also suggests that the environmental advantages of plant-based meat alternatives depend on multiple life cycle factors and production practices [44], underscoring the importance of a holistic assessment of dietary sustainability across multiple environmental dimensions.

Nutritionally, the reference scenario demonstrated that the Dutch adult population had a high level of vitamin B2 inadequacy [29], as the health risk associated with this level of intake and inadequacy was unclear; therefore, follow-up research on nutritional status should be performed. Additionally, women had consistently higher levels of inadequacy in vitamins B12, B6, and B2, and calcium, except for vitamin A, where the level of inadequacy was comparable between men and women (Supplementary Table S4). The replacement scenarios demonstrated various degrees of impact on the population’s nutrient intakes and adequacy, besides the well-documented increased fiber intake for all scenarios [13, 14, 45]. The ‘half meat’ scenario maintained overall nutrient adequacy, with no substantial increase in the risk of inadequacy, except that vitamin B6 inadequacy risk increased, particularly in women. In the ‘no meat’ scenario, the risk of vitamin B12 and B6 inadequacy increased, with additional reductions in total protein and saturated fat intake. Similar patterns were observed in the ‘no red meat’ scenario, as red meat contributed substantially (~ 80%) to total meat consumption (Supplementary Table S5). The most pronounced nutritional shifts occurred in the ‘no meat and dairy’ scenario, where inadequacy risks increased for vitamin B12, B6, B2, vitamin A, and calcium. Additionally, this scenario led to lower total protein intake but higher energy, total lipids (for equal weight replacement) intake, with reduced sodium intake. Our findings are consistent with recent reviews emphasizing that plant-based foods cannot fully replicate the nutritional profile of the animal-based counterparts [44, 45], highlighting the trade-offs between different replacement levels and the potential public health nutrition risks while shifting towards plant-based diets.

Uniquely in the Dutch context, we applied two functional units, i.e., weight (grams) and energy (kcal), to model replacement scenarios. Equal weight replacement is widely applicable in dietary modeling due to its simplicity [6, 7, 10, 11], while equal energy replacement provides a more physiologically relevant assessment of nutrient intakes. Unlike equal weight replacement, which may lead to unintended variations in energy intake due to differences in energy density between plant- and animal-based foods, equal energy replacement ensures that total caloric intake remains constant [46]. This prevents confounding effects on metabolism, satiety, and nutrient adequacy, making it a more robust approach for assessing the physiological impacts of dietary transitions [47]. It seems, according to our results, that equal weight replacement resulted in larger environmental benefits than equal energy replacement, while most nutritional effects remained comparable.

The study provides several policy and research insights. Our findings suggest that the ‘half meat’ scenario, which estimated involves a partial replacement of meat while maintaining current dairy consumption, represents a practical and achievable dietary transition that aligns with policy targets in the Netherlands while delivering environmental benefits. This strategy prioritizes replacing over elimination, making it a more feasible intermediate step toward a more sustainable diet. Notably, it aligns with flexitarian dietary practices [48] and maintains dairy consumption, which may also be more acceptable for Dutch consumers, where dairy products are culturally significant and widely consumed [49]. The health outcomes associated with dairy consumption are generally positive or neutral, whereas meat consumption is more frequently linked to adverse health effects [50–54]. Importantly, this transition preserves overall nutrient adequacy at the population level while contributing to environmental gains. However, careful attention should be given to the increased water footprint associated with plant-based protein sources.

Beyond nutritional and environmental considerations, consumer acceptance, cost and affordability, and accessibility of plant-based alternatives remain key factors influencing the successful adoption of dietary changes [55, 56]. In the Dutch context, evidence shows that, at the macro-level, consumer openness to plant-based foods is increasing, with a growing number of flexitarians and the rapid expansion of the plant-based product market in the retail environment [57, 58]. Furthermore, national initiatives (GR) and retailer commitments to promote plant-based protein consumption suggest that the proposed replacement scenarios are becoming increasingly feasible and culturally compatible [4, 57]. Future policies should consider these aspects to facilitate a sustainable and inclusive dietary transition. Future research could build on our modelling by assessing whether the modelled dietary changes support adherence to the Dutch dietary guidelines, and by extending the scenario modelling to more diverse population groups and sectors to evaluate how dietary transitions ultimately contribute to achieving targets for environmental impacts from diets [59].

Our dietary scenario modelling has several strengths that enhance its relevance to a sustainable dietary transition. First, we conducted dietary modeling at the whole-diet level, systematically replacing a wide range of meat and dairy products with a diverse selection of plant-based alternatives currently available in Dutch retail markets. This approach ensures that the modeled scenarios reflect realistic dietary shifts. Second, we utilized the most recent nationally representative dietary intake data from Dutch adults, along with national nutritional composition data and the latest life cycle assessment dataset (2024) tailored to the Dutch agri-food system. By evaluating nutrient adequacy at the population level based on estimated average intake, we demonstrated that while some micronutrient intakes were lower in replacement scenarios, they did not lead to an increased risk of inadequacy at the population level. Third, we assessed both environmental and nutritional outcomes concurrently, considering a broad range of environmental indicators (GHG, land use, and water footprint) alongside 13 key macro- and micro-nutrients. This comprehensive assessment provides a more holistic understanding of the trade-offs involved in dietary transitions. Lastly, we applied two distinct functional units, i.e., equal weight (mass-based) and equal energy (caloric-based) replacements, resulting in a total of eight dietary scenarios. Previous research has shown that the choice of functional unit substantially influences the sustainability performance of foods [60]. By incorporating both approaches, we addressed the methodological limitations inherent to each and provided a nuanced evaluation of sustainable dietary shifts.

Several limitations should be acknowledged. First, our modeling assumed an equal probability of replacement for plant-based alternatives corresponding to each food group, which may not fully reflect actual consumer preferences or market dynamics [61, 62]. Second, we did not account for variations in acceptance across different age and socio-demographic groups, which can influence the feasibility of dietary shifts. Future research should explore stratified analyses to better understand the adoption potential of plant-based diets among diverse population groups [63]. Third, our analysis did not consider differences in nutrient bioavailability between animal- and plant-based protein sources. While plant-based diets can provide adequate nutrient intakes, factors such as lower bioavailability of certain micronutrients (e.g., iron, zinc, and vitamin B12) could impact long-term nutritional status [64]. Fourth, we estimated habitual intake and the associated risk of inadequacy at the population level but could not determine precisely which individuals or subgroups might be at greater risk. Nutrient needs vary based on factors such as age, sex, and physiological status, which were not explicitly accounted for in this study. Fifth, like all studies relying on self-reported dietary intake data, our analysis is subject to reporting errors, including potential underreporting of food intake [65–67]. This could lead to an underestimation of absolute nutrient intake levels, though relative differences between scenarios remain informative. Future studies incorporating objective biomarkers or alternative dietary assessment methods could strengthen the accuracy of dietary intake estimations. Lastly, some meat and dairy alternatives included as replacement foods were fortified with vitamin B12 and calcium, respectively. Therefore, the risk of inadequacies of vitamin B12 and calcium might be slightly underestimated if plant-based alternatives were not fortified. Besides the impact on risk of inadequacies, the environmental impact of food fortification should also be evaluated, a limitation of this study is that we could not explicitly model the effects of fortification or supplementation, which may be important strategies to mitigate potential nutrient inadequacies. These limitations emphasize the need for cautious interpretation of the results and suggest that future studies incorporating objective nutrient biomarkers, and populations with low/no consumption of meat and dairy could further strengthen understanding of the impacts of plant-based dietary shifts.

## Conclusion

This study assessed the sustainability and nutritional impacts of replacing meat and dairy with plant-based alternatives in the Netherlands. All replacement scenarios reduced GHG and land use but increased water footprint. The “half meat” scenario met the national protein transition target while maintaining dairy consumption and overall nutrient adequacy, whereas more extensive replacements heightened the plant-based protein over the animal-based protein intake as well as the risk of micronutrient inadequacy. These findings suggest that gradual, flexitarian shifts offer a more feasible path toward sustainable diets. Future research should address consumer acceptance, cost and affordability, and nutrient bioavailability to support effective dietary transitions.

## Supplementary Information

Below is the link to the electronic supplementary material.

Supplementary Material 1

## Data Availability

The authors do not have the right to share the datasets used in this study. The Dutch Ministry of Health, Welfare and Sports is the formal owner of DNFCS, while the National Institute for Public Health and the Environment (RIVM) takes care of data management and data distribution to other users. However, data of this survey is available upon request (https://www.rivm.nl/en/dutch-national-food-consumption-survey/data-on-request). NEVO data are accessible and can be downloaded from a website: https://www.rivm.nl/en/dutch-food-composition-database/nevo-online-request-dataset. The detailed LCA dataset is internally available within RIVM as a web application. The DISC-tool is also internally available within RIVM as a R Shiny web application or as the code and can be made available upon request. SPADE is an open source and downloadable R package via the RIVM website: https://www.rivm.nl/en/spade/access-to-spade.
