# Supplementary material for: Replacing meat and dairy with plant-based alternatives in the Netherlands: trade-offs in environmental impacts and critical nutrient intake
Source: Eur J Nutr. 2026 Feb 16;65(2):62. doi: 10.1007/s00394-026-03908-w (PMC12909465; doi:10.1007/s00394-026-03908-w)
Supplement: Supplementary file 1 — Supplementary Material 1 [file 394_2026_3908_MOESM1_ESM.docx]

**Supplementary Material**

**Replacing meat and dairy products with plant-based alternatives in the Netherlands: trade-offs in environmental impacts and critical nutrient intake**

Zhu *et al.*

**Table of Contents**

[Supplementary Table S2. Habitual energy and nutrient intakes (95% CI) in reference and replacement scenarios in women and men aged 18-65 years. 3](#_Toc195782785)

[Supplementary Table S3. Average consumption of food groups to be replaced at DNFCS 2019-2021 (reference scenario) in women and men aged 18-65 years. 5](#_Toc195782786)

[Supplementary Table S4. Population at risk of nutrient inadequacy (95% CI) in reference and replacement scenarios in women and men aged 18-65 years. 6](#_Toc195782787)

[Supplementary Table S5. Environmental indicators of reference and replacement scenarios in adults aged 18-65 years. 7](#_Toc195782788)

[Supplementary Table S6. Average environmental impact indicators of the consumption of food groups to be replaced at DNFCS 2019-2021. 8](#_Toc195782789)

[Figure S1. Habitual total protein intake in adults aged 18-65 years in reference and replacement scenarios in women and men. 9](#_Toc195782790)

[Figure S2. Habitual plant-based and animal-based protein intake in adults aged 18-65 years in reference and replacement scenarios in women and men. 10](#_Toc195782791)

[Figure S3. Habitual intake of total energy in adults aged 18-65 years in reference and replacement scenarios in women and men. 11](#_Toc195782792)

[Figure S4. Habitual intake of total lipids in adults aged 18-65 years in reference and replacement scenarios in women and men. 12](#_Toc195782793)

[Figure S5. Habitual intake of saturated fatty acids (SAFA) in adults aged 18-65 years in reference and replacement scenarios in women and men. 13](#_Toc195782794)

[Figure S6. Habitual intake of fiber in adults aged 18-65 years in reference and replacement scenarios in women and men. 14](#_Toc195782795)

[Figure S7. Habitual intake of sodium in adults aged 18-65 years in reference and replacement scenarios in women and men. 15](#_Toc195782796)

[Figure S8. Greenhouse gas emission (GHG), in adults aged 18-65 in reference and replacement scenarios. 16](#_Toc195782797)

[References 17](#_Toc195782798)

Supplementary Table S1. Estimated Average Requirement (EAR) of nutrient intakes in men and women aged 18-65 years.

| Nutrients | Men | Women |
| --- | --- | --- |
| Vitamin A (retinol activity equivalent), μg/day | 615 | 530 |
| Vitamin B12, μg/day | 2 | 2 |
| Vitamin B6, mg/day | 1.1 | 1.1 |
| Vitamin B2, mg/day | 1.3 | 1.3 |
| Calcium, mg/day | 750 | 750 |

* EAR was based on the values reported by the Health Council of the Netherlands (1, 2).

# Supplementary Table S2. Habitual energy and nutrient intakes (95% CI) in reference and replacement scenarios in women and men aged 18-65 years.

|  |  | Women | | | | | | | |
| --- | --- | --- | --- | --- | --- | --- | --- | --- | --- |
|  | Reference | No meat and dairy | | No meat | | Half meat | | No red meat | |
|  |  | Equal weight | Equal energy | Equal weight | Equal energy | Equal weight | Equal energy | Equal weight | Equal energy |
| Total energy, kcal/day | 1844.9 (1801.9-1890.8) | 1966.7 (1921.3-2015.9) | 1844.9 (1801.9-1890.8) | 1853 (1809.1-1900.9) | 1844.9 (1801.9-1890.8) | 1853.5 (1810.3-1900.1) | 1844.9 (1801.9-1890.8) | 1848 (1804.1-1895) | 1844.9 (1801.9-1890.8) |
| Total protein, g/day | 71 (69.1-72.8) | 56.7 (55.3-58.2) | 58.9 (57.3-60.6) | 62.9 (61.4-64.7) | 66.1 (64.3-67.9) | 66.8 (65.2-68.6) | 68.2 (66.4-70.1) | 66.2 (64.5-68) | 68.8 (67-70.7) |
| Animal-based protein, g/day | 42.7 (41.2-44.5) | 11.7 (10.6-12.8) | 12.1 (11-13.3) | 26.9 (25.7-28.2) | 27.2 (26-28.5) | 35.1 (33.8-36.6) | 35.2 (33.9-36.7) | 32.4 (31-33.8) | 32.7 (31.3-34.1) |
| Plant-based protein, g/day | 29.1 (28.2-30.1) | 48.2 (47-49.5) | 50.4 (48.9-51.8) | 37.4 (36.6-38.7) | 40.9 (39.6-42.3) | 33.4 (32.5-34.5) | 35.2 (34-36.5) | 35.5 (34.6-36.5) | 38.6 (37.3-39.9) |
| Total lipids, g/day | 80.5 (78.2-83.1) | 94.7 (91.9-97.7) | 78.9 (76.7-81.5) | 80.8 (78.3-83.6) | 76.5 (74.3-79) | 81.2 (78.9-83.8) | 78.7 (76.5-81.2) | 80 (77.6-82.7) | 77.1 (74.8-79.6) |
| SAFA, g/day | 29.3 (28.4-30.4) | 27.9 (26.6-29.4) | 21.5 (20.7-22.3) | 26.7 (25.8-27.8) | 26.2 (25.3-27.2) | 28.2 (27.3-29.2) | 27.9 (27-28.9) | 26.9 (26-28) | 26.5 (25.6-27.6) |
| Fiber, g/day | 19.6 (19-20.2) | 27.1 (26.5-27.9) | 28.7 (27.8-29.6) | 23.7 (23.1-24.3) | 25.9 (25.2-26.9) | 21.7 (21.1-22.3) | 22.8 (22.1-23.6) | 22.3 (21.8-23) | 24.1 (23.4-24.9) |
| Vitamin A*, μg/day | 811 (754-878) | 501 (464-544) | 501 (465-544) | 643 (608-679) | 644 (610-681) | 725 (680-780) | 726 (681-781) | 647 (613-685) | 648 (615-686) |
| Vitamin B12, μg/day | 3.9 (3.7-4.2) | 2.3 (2.1-2.5) | 2.8 (2.5-3) | 3 (2.9-3.2) | 3.1 (2.9-3.3) | 3.5 (3.3-3.7) | 3.5 (3.3-3.7) | 3.1 (2.9-3.3) | 3.2 (3-3.3) |
| Vitamin B6, mg/day | 1.4 (1.4-1.5) | 1.2 (1.2-1.3) | 1.2 (1.2-1.2) | 1.2 (1.2-1.2) | 1.2 (1.2-1.2) | 1.3 (1.3-1.4) | 1.3 (1.3-1.4) | 1.3 (1.3-1.4) | 1.3 (1.3-1.3) |
| Vitamin B2, mg/day | 1.3 (1.2-1.3) | 1 (1-1.1) | 1.2 (1.1-1.3) | 1.2 (1.2-1.3) | 1.3 (1.2-1.3) | 1.3 (1.2-1.3) | 1.3 (1.2-1.3) | 1.2 (1.2-1.3) | 1.3 (1.2-1.3) |
| Calcium, mg/day | 953 (921-987) | 709 (687-732) | 843 (809-878) | 1012 (979-1047) | 1043 (1007-1078) | 981 (949-1015) | 993 (960-1028) | 1000 (967-1036) | 1028 (993-1064) |
| Sodium, mg/day | 2146 (2090-2214) | 1834 (1781-1893) | 1940 (1887-2000) | 1978 (1926-2039) | 2059 (2003-2116) | 2060 (2002-2120) | 2094 (2034-2155) | 1995 (1939-2054) | 2068 (2012-2127) |
|  |  | Men | | | | | | | |
|  | Reference | No meat and dairy | | No meat | | Half meat | | No red meat | |
|  |  | Equal weight | Equal energy | Equal weight | Equal energy | Equal weight | Equal energy | Equal weight | Equal energy |
| Total energy, kcal/day | 2439.2 (2377.5-2497) | 2576.4 (2512.9-2637.2) | 2439.2 (2377.5-2497) | 2443.7 (2382.5-2501.1) | 2439.2 (2377.5-2497) | 2449 (2386.8-2507) | 2439.2 (2377.5-2497) | 2438.4 (2376.2-2496.7) | 2439.2 (2377.5-2497) |
| Total protein, g/day | 95.4 (92.5-98) | 73.9 (71.9-75.9) | 79.3 (76.8-81.4) | 81.8 (79.6-83.8) | 87.7 (85.2-90.2) | 88.9 (86.1-91.3) | 91.5 (88.6-94) | 87.7 (84.9-90.4) | 92.4 (89.4-95) |
| Animal-based protein, g/day | 57.8 (55.2-60.3) | 13.2 (11.4-15) | 13.9 (12-15.9) | 30.8 (29.4-32.2) | 31.3 (29.9-32.7) | 45 (42.8-47) | 45.2 (43-47.2) | 40.8 (38.6-43) | 41.2 (39-43.4) |
| Plant-based protein, g/day | 39.1 (37.7-40.3) | 65.1 (63.3-66.7) | 70.1 (67.8-72.2) | 52.7 (51.1-54.2) | 58.9 (56.6-60.8) | 46.1 (44.7-47.5) | 49.4 (47.5-50.9) | 49.1 (47.7-50.4) | 54.5 (52.5-56.1) |
| Total lipids, g/day | 104 (100.8-107) | 119.4 (115.8-122.8) | 100.2 (97.3-102.9) | 102.8 (99.6-105.7) | 96.8 (93.9-99.5) | 104.4 (101.1-107.4) | 100.9 (97.8-103.8) | 102.2 (99-105.2) | 97.9 (94.9-100.7) |
| SAFA, g/day | 36.6 (35.4-37.7) | 34.7 (33.1-36.3) | 26.3 (25.3-27.2) | 32.4 (31.2-33.4) | 31.5 (30.3-32.5) | 34.7 (33.5-35.8) | 34.3 (33.1-35.3) | 32.7 (31.5-33.7) | 32 (30.9-33) |
| Fiber, g/day | 24.9 (24.1-25.8) | 35.5 (34.3-36.5) | 38.6 (37.1-39.9) | 31.5 (30.4-32.6) | 35.4 (34-36.8) | 28.4 (27.4-29.3) | 30.4 (29.3-31.5) | 29.3 (28.4-30.2) | 32.3 (31.2-33.4) |
| Vitamin A*, μg/day | 972 (890-1050) | 544 (501-583) | 545 (502-584) | 716 (672-757) | 718 (674-759) | 820 (763-872) | 821 (764-874) | 726 (682-767) | 728 (684-769) |
| Vitamin B12, μg/day | 5.2 (4.8-5.6) | 2.7 (2.4-3) | 3.2 (2.9-3.5) | 3.7 (3.4-3.9) | 3.7 (3.4-4) | 4.4 (4.1-4.7) | 4.4 (4.1-4.7) | 3.8 (3.5-4.1) | 3.8 (3.6-4.1) |
| Vitamin B6, mg/day | 1.9 (1.8-2) | 1.5 (1.5-1.6) | 1.5 (1.4-1.5) | 1.5 (1.4-1.5) | 1.5 (1.4-1.5) | 1.7 (1.6-1.8) | 1.7 (1.6-1.8) | 1.7 (1.6-1.8) | 1.7 (1.6-1.8) |
| Vitamin B2, mg/day | 1.6 (1.6-1.7) | 1.3 (1.3-1.4) | 1.5 (1.5-1.6) | 1.6 (1.5-1.6) | 1.6 (1.5-1.7) | 1.6 (1.5-1.6) | 1.6 (1.6-1.7) | 1.6 (1.5-1.6) | 1.6 (1.5-1.7) |
| Calcium, mg/day | 1136 (1102-1175) | 853 (823-883) | 1044 (994-1090) | 1232 (1198-1272) | 1282 (1245-1326) | 1187 (1152-1228) | 1214 (1177-1258) | 1212 (1179-1252) | 1259 (1223-1303) |
| Sodium, mg/day | 2734 (2662-2806) | 2307 (2240-2373) | 2474 (2401-2545) | 2473 (2406-2539) | 2597 (2523-2668) | 2596 (2528-2662) | 2655 (2584-2724) | 2498 (2430-2563) | 2614 (2540-2684) |

*as retinol activity equivalent

# Supplementary Table S3. Average consumption of food groups to be replaced at DNFCS 2019-2021 (reference scenario) in women and men aged 18-65 years.

| Food groups | Women (g/day) | Men (g/day) | Women (kcal/day) | Men (kcal/day) |
| --- | --- | --- | --- | --- |
| Milk drink, non-sweetened | 129.9 | 187.5 | 57.4 | 83.3 |
| Milk drink, sweetened | 40.3 | 48.2 | 18.8 | 24.1 |
| Coffee milk | 3.4 | 3.8 | 4.1 | 4.3 |
| Dairy dessert | 33.0 | 49.1 | 49.5 | 68.7 |
| Fermented dairy products | 129.8 | 127.7 | 87.8 | 85.0 |
| Cream products | 4.2 | 5.6 | 11.6 | 15.8 |
| Cheese | 38.8 | 44.0 | 131.3 | 153.5 |
| Cheese spread | 6.5 | 3.6 | 16.4 | 9.3 |
| Cheese-based warm snacks | 2.2 | 1.7 | 8.3 | 7.7 |
| Meat unprocessed | 51.3 | 77.2 | 108 | 164.6 |
| Meat processed | 29.0 | 45.4 | 94.1 | 145.9 |
| Meat-based warm snacks | 13.0 | 19.2 | 36.2 | 52.3 |
| Cold meat spread | 1.8 | 4.3 | 5.6 | 13.3 |
| Cold meat cut | 20.8 | 30.1 | 47.9 | 74.6 |
| Total dairy | 388.1 | 471.2 | 385.2 | 451.7 |
| Total meat | 115.9 | 176.2 | 291.8 | 450.7 |
| Total red meat | 91.8 | 143.4 | 249.9 | 393.5 |

# Supplementary Table S4. Population at risk of nutrient inadequacy (95% CI) in reference and replacement scenarios in women and men aged 18-65 years.

|  |  | Women | | | | | | | |
| --- | --- | --- | --- | --- | --- | --- | --- | --- | --- |
|  | Reference | No meat and dairy | | No meat | | Half meat | | No red meat | |
|  |  | Equal weight | Equal energy | Equal weight | Equal energy | Equal weight | Equal energy | Equal weight | Equal energy |
| Vitamin B12 | 7.3 (4.2-10.4) | 41.9 (34.5-50.4) | 28.1 (20.8-36.1) | 21.9 (16.7-27) | 20.5 (15.5-25.6) | 11.9 (7.9-16.5) | 11.8 (7.8-16.2) | 20.5 (15.7-25.5) | 19.5 (14.7-24.3) |
| Vitamin B6 | 14.7 (10.2-18.6) | 37.2 (32.9-41.9) | 40.8 (36.6-45.4) | 41.1 (37.1-45.6) | 41.9 (37.9-46.4) | 24.4 (19.5-28.9) | 24.7 (19.8-29.3) | 26.7 (21.9-31.3) | 27.2 (22.4-31.7) |
| Vitamin B2 | 56.5 (52.3-60.7) | 80.1 (76.2-83.7) | 65.2 (60-70.6) | 61.1 (56.9-65.1) | 59.3 (55.2-63.6) | 58.5 (54.4-62.8) | 57.9 (53.7-62.3) | 61 (56.8-65) | 59 (54.9-63.3) |
| Calcium | 28.2 (23.7-32.6) | 64.3 (59.1-68.2) | 42.1 (36.7-47.8) | 21.9 (17.5-26.1) | 19.4 (15.2-23.7) | 24.9 (20.5-29.2) | 23.8 (19.4-28.2) | 23.3 (18.8-27.6) | 21 (16.5-25.3) |
| Vitamin A* | 19.7 (12.8-25.6) | 62.5 (53.9-69.6) | 67.7 (61.6-74.3) | 32.6 (25.3-39.5) | 32.1 (24.8-39) | 23.2 (16-29.5) | 23 (15.8-29.3) | 31.7 (24.3-38.6) | 31.5 (24-38.3) |
|  |  | Men | | | | | | | |
|  | Reference | No meat and dairy | | No meat | | Half meat | | No red meat | |
|  |  | Equal weight | Equal energy | Equal weight | Equal energy | Equal weight | Equal energy | Equal weight | Equal energy |
| Vitamin B12 | 1 (0-2.2) | 31.3 (23.5-39.3) | 19.6 (12.9-26) | 9.7 (5.5-14.1) | 8.9 (4.9-13.2) | 2.3 (0.4-4.4) | 2.1 (0.3-4.1) | 9.2 (5.3-13.5) | 8.6 (4.9-12.8) |
| Vitamin B6 | 5.9 (3.6-8.2) | 22 (18-25.9) | 23.2 (19.1-27) | 24.3 (20.3-28) | 24.5 (20.3-28.1) | 12.6 (9.3-16.1) | 12.7 (9.5-16.2) | 13.7 (10.6-16.9) | 13.7 (10.6-17) |
| Vitamin B2 | 27.4 (23.3-31.4) | 53.6 (48.3-58.8) | 37.9 (31.2-44.1) | 33.5 (29.2-37.6) | 30.9 (26.4-35.1) | 30.4 (26.2-34.6) | 30 (25.9-34.2) | 33.3 (29.1-37.3) | 30.7 (26.2-35) |
| Calcium | 12.1 (7.4-16.6) | 37.1 (31.9-42.2) | 18.8 (13-24.4) | 6.4 (3.3-9.5) | 4.8 (2.2-7.4) | 8.7 (4.7-12.7) | 7.6 (3.9-11.3) | 7.3 (3.9-10.7) | 5.4 (2.4-8.2) |
| Vitamin A* | 21.7 (14.8-28.5) | 67.8 (61.8-74.5) | 62.4 (53.8-69.4) | 38.1 (31.6-45.5) | 37.9 (31.4-45.1) | 28.3 (21.7-35.5) | 28.1 (21.4-35.2) | 36.3 (29.6-43.7) | 36 (29.3-43.4) |

*as retinol activity equivalent

# Supplementary Table S5. Environmental indicators of reference and replacement scenarios in adults aged 18-65 years.

| Scenario case | Replacement strategy | GHG (kg CO₂-eq/day) | Land use (m²·year/day) | Water footprint (liters/day) |
| --- | --- | --- | --- | --- |
| Reference |  | 3.99 | 3.30 | 95.82 |
| No meat and dairy | Equal weight | 2.42 | 2.99 | 153.55 |
|  | Equal energy | 2.48 | 2.80 | 124.42 |
| No meat | Equal weight | 3.02 | 2.71 | 115.76 |
|  | Equal energy | 3.10 | 2.79 | 101.44 |
| Half meat | Equal weight | 3.50 | 3.02 | 107.12 |
|  | Equal energy | 3.54 | 3.05 | 99.32 |
| No red meat | Equal weight | 3.08 | 2.79 | 111.28 |
|  | Equal energy | 3.16 | 2.85 | 100.70 |

# Supplementary Table S6. Average environmental impact indicators of the consumption of food groups to be replaced at DNFCS 2019-2021.

| Food groups | GHG (kg CO₂-eq/day) | Land use (m²·year/day) | Water footprint (liters/day) |
| --- | --- | --- | --- |
| Milk drink, non-sweetened | 0.23 | 0.093 | 1.85 |
| Milk drink, sweetened | 0.075 | 0.032 | 0.6 |
| Coffee milk | 0.006 | 0.0024 | 0.051 |
| Dairy dessert | 0.1 | 0.047 | 0.83 |
| Fermented dairy products | 0.27 | 0.13 | 11 |
| Cream products | 0.023 | 0.012 | 0.17 |
| Cheese | 0.39 | 0.18 | 2.98 |
| Cheese spread | 0.025 | 0.011 | 0.21 |
| Cheese-based warm snacks | 0.007 | 0.0065 | 0.046 |
| Meat unprocessed | 0.91 | 0.69 | 6.24 |
| Meat processed | 0.61 | 0.44 | 3.8 |
| Meat-based warm snacks | 0.074 | 0.074 | 0.56 |
| Cold meat spread | 0.015 | 0.013 | 0.1 |
| Cold meat cut | 0.27 | 0.23 | 1.76 |
| Total dairy | 1.126 | 0.5139 | 17.737 |
| Total meat | 1.879 | 1.447 | 12.46 |
| Total red meat | 1.819 | 1.108 | 11.15 |


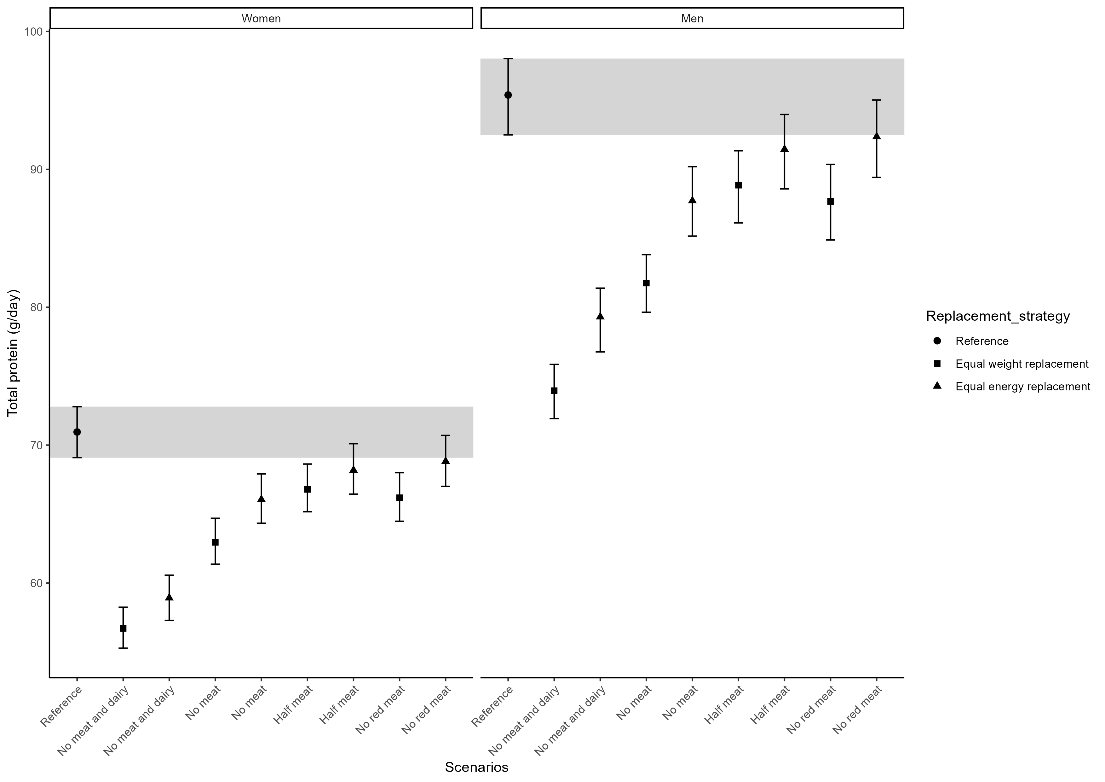


Figure S1. Habitual total protein intake (g/day) in adults aged 18-65 years in reference and replacement scenarios in women and men. The intervals lines represent 95% confidence interval derived from 200 bootstrap resamples for each scenarios.


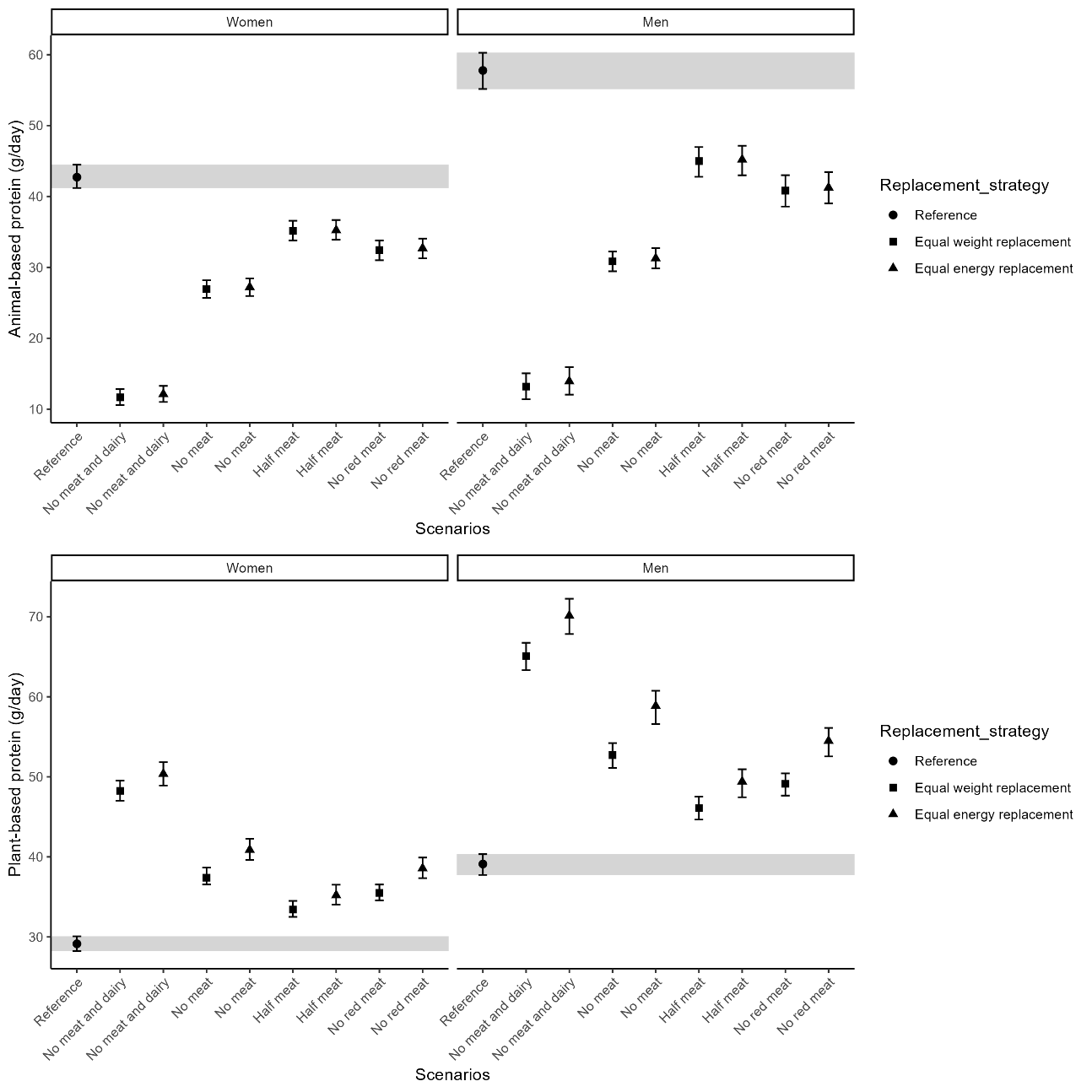


# Figure S2. Habitual plant-based and animal-based protein intake (g/day) in adults aged 18-65 years in reference and replacement scenarios in women and men. The intervals lines represent 95% confidence interval derived from 200 bootstrap resamples for each scenarios.


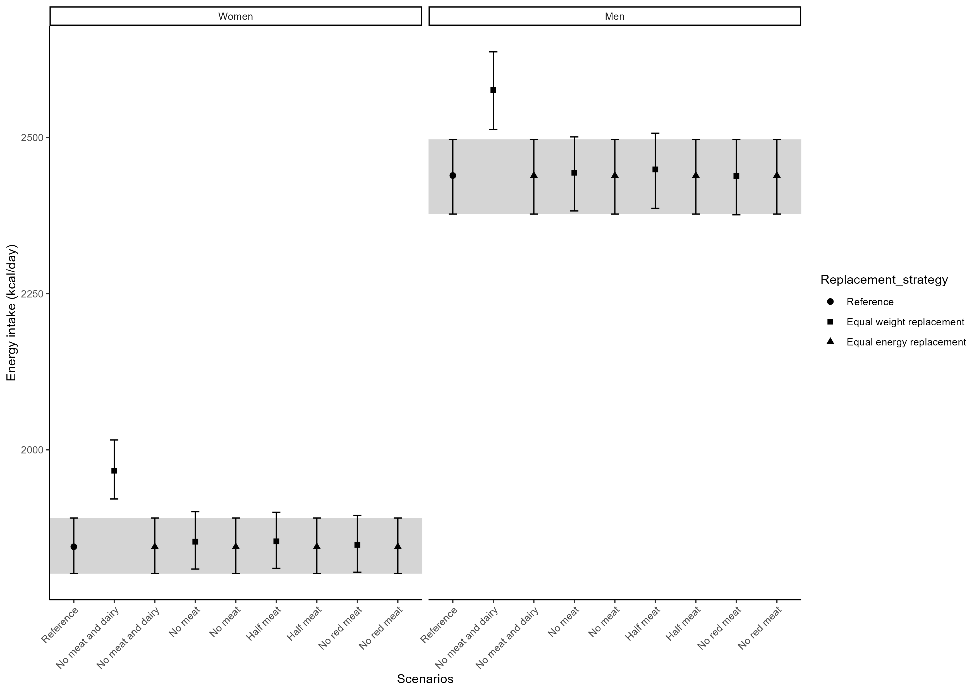


# Figure S3. Habitual intake of total energy (kcal/day) in adults aged 18-65 years in reference and replacement scenarios in women and men. The intervals lines represent 95% confidence interval derived from 200 bootstrap resamples for each scenarios.


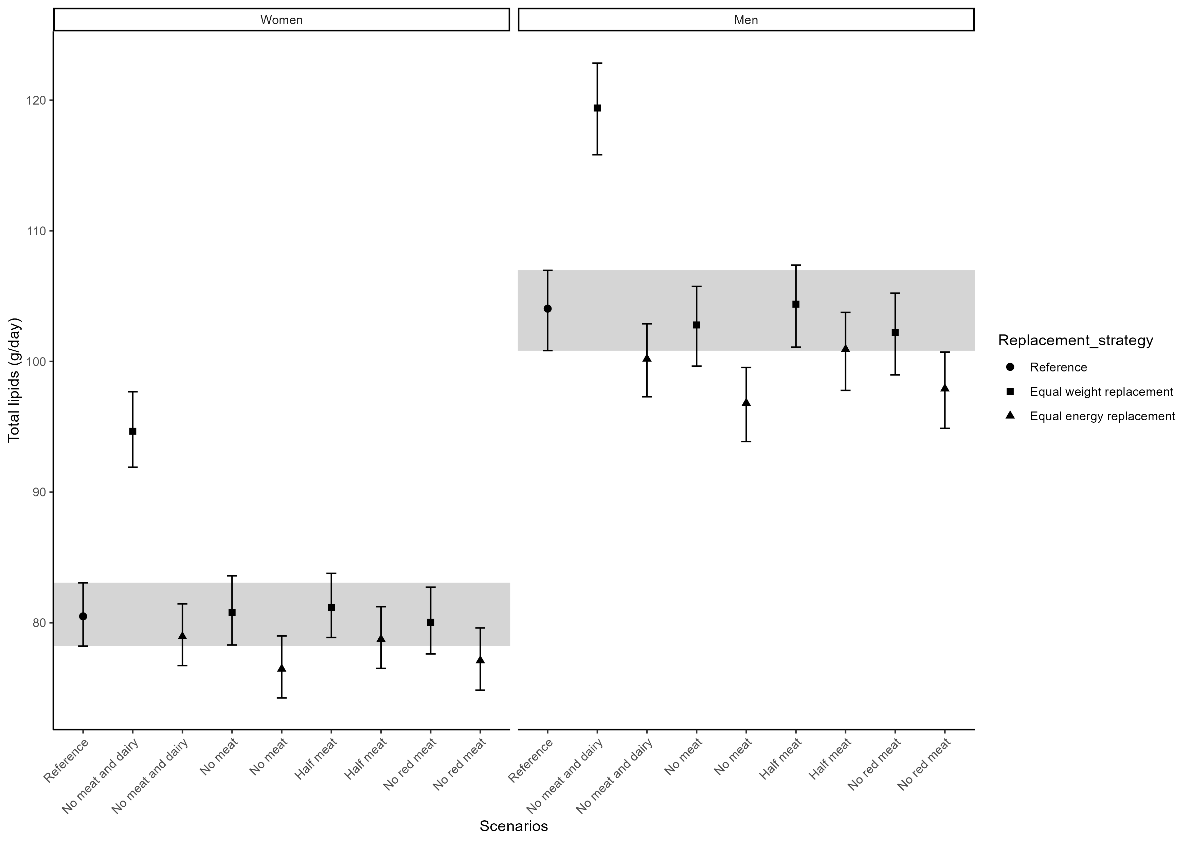


# Figure S4. Habitual intake of total lipids (g/day) in adults aged 18-65 years in reference and replacement scenarios in women and men. The intervals lines represent 95% confidence interval derived from 200 bootstrap resamples for each scenarios.


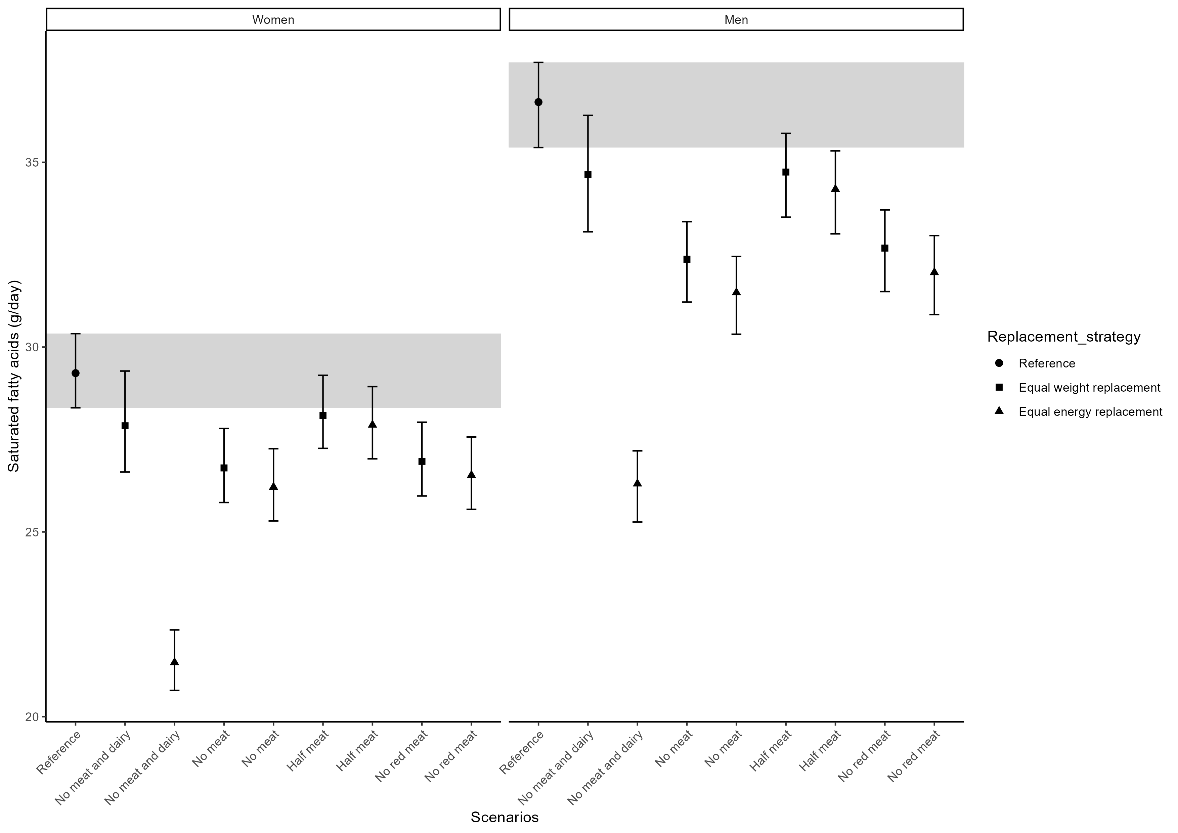


# Figure S5. Habitual intake of saturated fatty acids (SAFA, g/day) in adults aged 18-65 years in reference and replacement scenarios in women and men. The intervals lines represent 95% confidence interval derived from 200 bootstrap resamples for each scenarios.


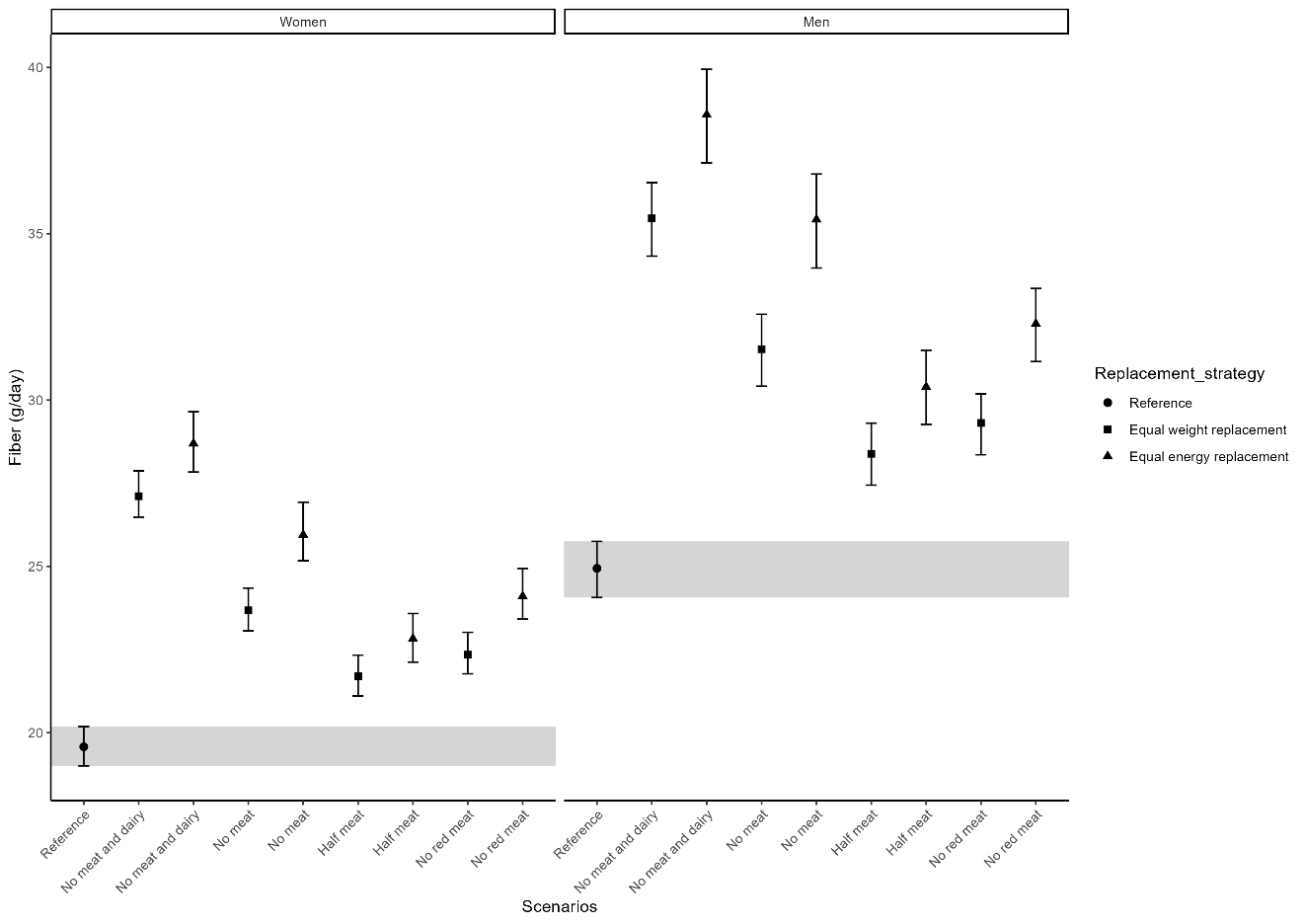


# Figure S6. Habitual intake of fiber (g/day) in adults aged 18-65 years in reference and replacement scenarios in women and men. The intervals lines represent 95% confidence interval derived from 200 bootstrap resamples for each scenarios.


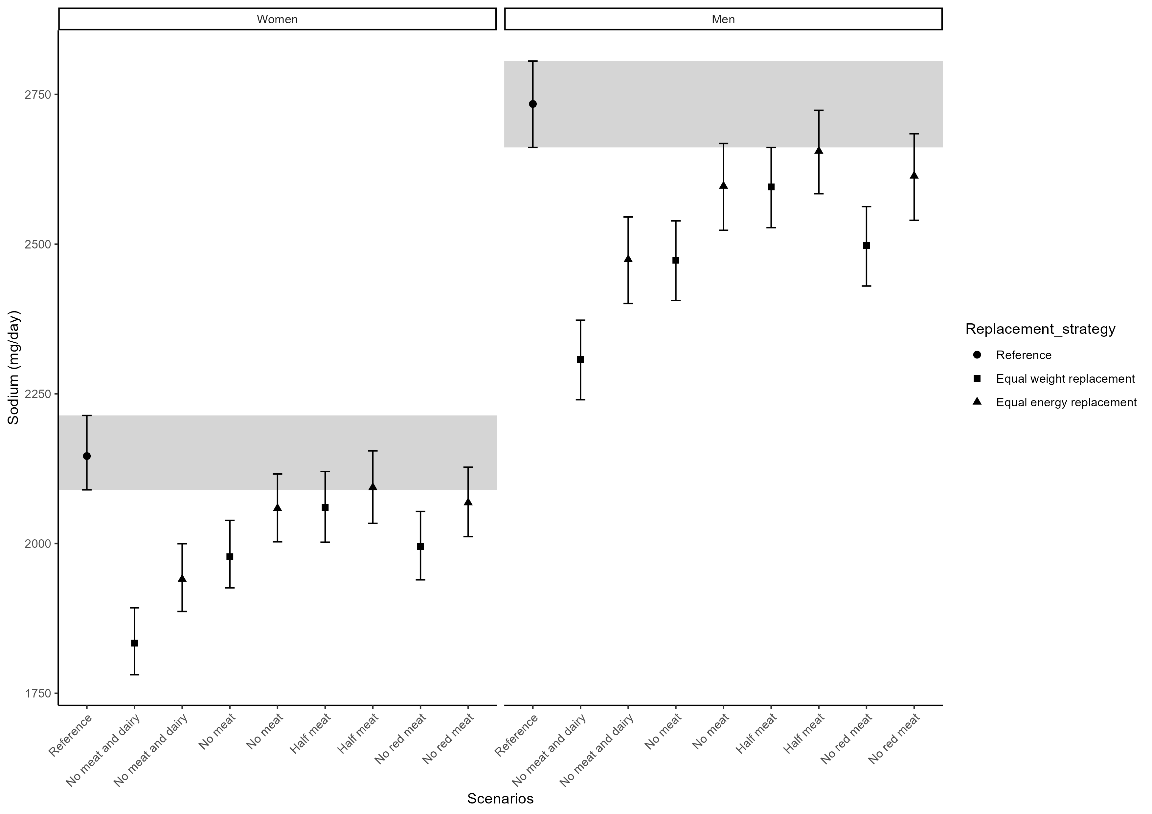


# Figure S7. Habitual intake of sodium (mg/day) in adults aged 18-65 years in reference and replacement scenarios in women and men. The intervals lines represent 95% confidence interval derived from 200 bootstrap resamples for each scenarios.


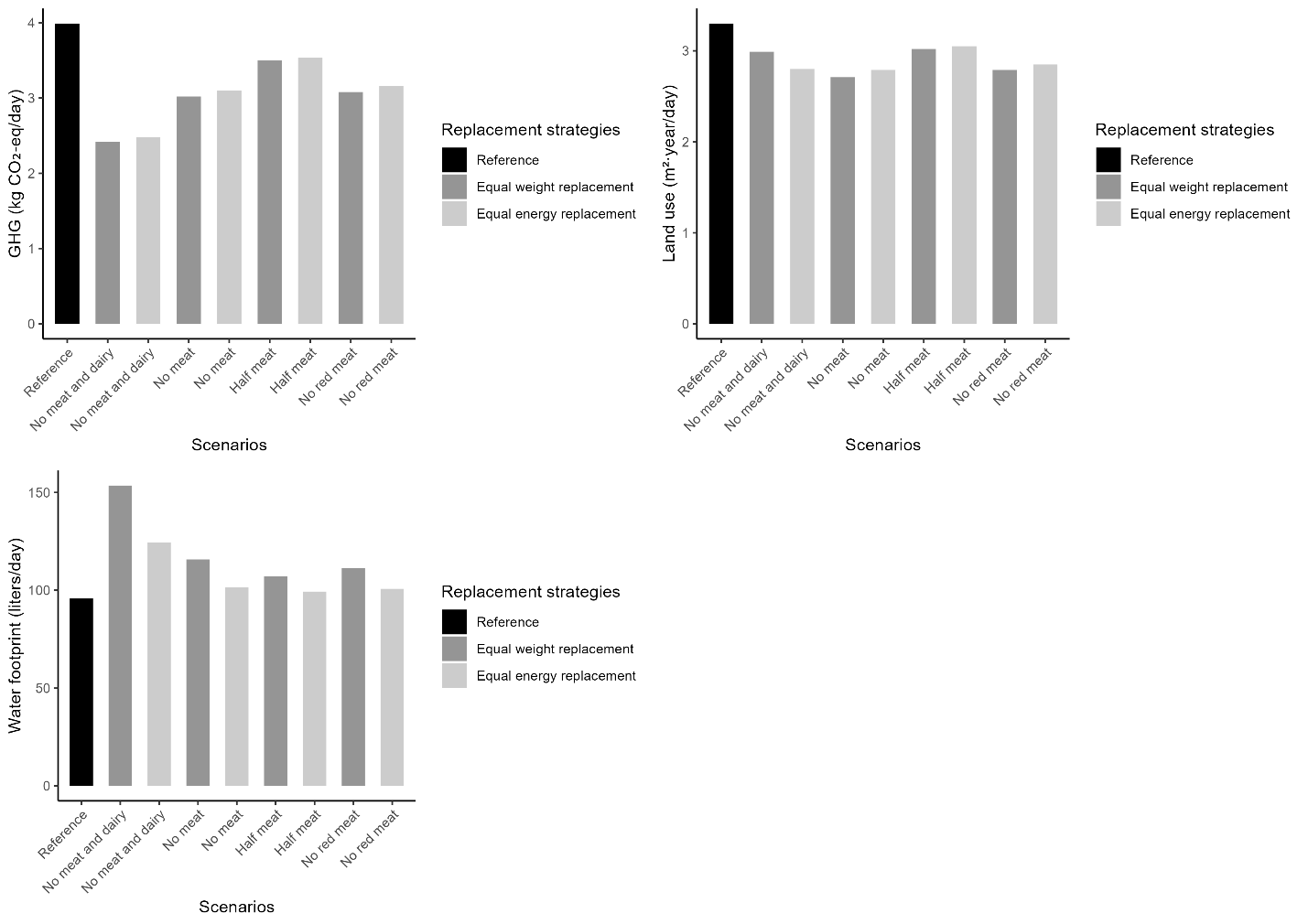


# Figure S8. Greenhouse gas emission (GHG, kg CO₂-eq/day), land use (m²·year/day), and water footprint (liters/day) in adults aged 18-65 in reference and replacement scenarios.

# References

1. Dietary reference values for vitamins and minerals for adults. The Hague: Health Council of the Netherlands; 2018. Contract No.: 2018/19e.

2. Sanderman-Nawijn EL, Brants HAM, Dinnissen CS, Ocké MC, van Rossum CTM. Energy and nutrient intake in the Netherlands - Results of the Dutch National Food Consumption Survey 2019-2021. Bilthoven: National Institute for Public Health and the Environment, RIVM; 2024. Contract No.: 2024-0071.
